# Progress in Medicine

**Published:** 1898-01-01

**Authors:** 


### Medical Progress and Hospital Clinics.

[*The Editor will be glad to receive offers of co-operation and contributions from members of the profession. All letters should be addressed to* The Editor, at the Office, 28 & 29, Southampton Street, Strand, London, W.C.]

### Progress in Medicine.

### FEVERS.

(*Continued from page* 224.)

**Small-pox.**—It is interesting to note the arguments for vaccination which seem to have had most weight with the legal mind of Lord Herschell.[1] As head of the Royal Commission, he had to digest and analyse the evidence for his colleagues, and he confesses that he was surprised at the strength of the proofs brought forward on behalf of vaccination. Thus, while noticing that the protection afforded is most efficient in the first nine or ten years, he contrasts the mortality of children in this country before and after the compulsory practice of vaccination. That mortality was reduced from 1,514 per million to 50. At the same time, the mortality of those in later life (who escaped the compulsory law) only fell from 24 to 19 per million. Again, in the epidemics of Sheffield and Warrington, the proportion of the deaths of children, who are most protected by vaccination, to those of adults was 25 to 100; while in Leicester and Gloucester it rose to 60 or 70. The former towns were well vaccinated, and the latter not. Once more, the deaths of vaccinated children in six epidemics were at the outside only 2·8 per cent. of those attacked, but the mortality in the unvaccinated rose to 30·3 per cent. of the attacks. Such facts as these, he adds, should be pointedly called to public attention. Yet these are only a small part of the case. Milnes argued that vaccination and small-pox attained their maximum together, and together they have steadily declined; but the *British Medical Journal*[2] points out that all recent epidemics have been modified by the once universal infant vaccination, and that among children, as vaccination has been neglected, so has the child mortality from small-pox increased. Moreover, the argument that, because fever and cholera have decreased, the diminution of small-pox cannot be due to vaccination will not hold water, because these diseases may be communicated through filth and defective water supplies, while small-pox arises through direct exposure to infection only. Mr. Milnes, too, asserts that males are more often revaccinated than females, instancing the army and postal service (though these are a small fraction of the population), and yet they suffer more from small-pox than females. But they are exposed to infection far more than females, and reckoning the numbers of female servants revaccinated it is very doubtful whether there is any excess of male protection.

In Germany it has been found possible to dilute calf lymph with glycerine to such an extent that one calf will supply enough to vaccinate 6,000 or more individuals. Tubercle and pus-forming organisms are destroyed by the addition of glycerine to the lymph while the activity of the vaccine is unaffected. Thus various diseases are prevented, while the amount of vaccine to be obtained from each calf is enormously increased, and can be stored without weakening for a month or longer. Sir R. Thorne Thorne therefore advises the Local Government Board that all State vaccination should be performed with calf lymph.[3] The preparation of the lymph on the continent is carried out in laboratories with the most elaborate precautions, which must be reproduced in this country if we are to meet the natural fears of parents. Cassenko[4] reports great success in the treatment of small-pox from inunction with icthyol ointment of the strength of one part in nine. This was rubbed in three times a day as soon as the papules became visible. The low temperature, rapid desquamation, and absence of inflammation which followed this application were very marked, so that the treatment merits further trials.

**Tropical Diseases.**—A strong claim has been made for special teaching in this subject by P. Manson,[5] and with good reason, for while more than a fifth of the profession practise in the tropics, and a great part of the population of the empire dwell there, there are numbers of fatal diseases peculiar to those climates which demand special knowledge and methods of treatment. "Experience," as Davidson remarks, "is a good teacher, only the fees are very heavy." Thus malaria, beri-beri, filiaria, and anæmia produced by ankylostomum destroy great multitudes every year, and yet there is hardly any teaching as to their diagnosis and as to recent successful methods of treating such diseases. Isolated cases are constantly turning up at home in returned sailors and wanderers, and are rarely diagnosed. It is difficult to estimate the loss of life in our West Indian islands from ignorance of the simple fact that thymol completely cures their pernicious anæmia. Even malaria, which is common enough in returned travellers, and perhaps in a few localities at home, is at times most difficult to diagnose. It may cause, *e.g.*, an apoplectic attack without fever, and it is constantly mistaken for typhoid. Osler[6] finds in the States three forms—tertian, which, however, may cause attacks daily or every other day; the quartan, which may also exist in double crops; and the irregular pernicious form. It is important for the diagnosis of simple malaria to note the intermittent pyrexia, which rarely lasts less than eleven or more than twelve hours. It differs in this from septicæmia, typhoid, tuberculosis, abscess of the liver, and gall-stone fever. Moreover, there is no increase of white corpuscles; the hæmatozoon is found in the blood in the simple forms, though less easily in the severe ones. There is anæmia and quick reaction to quinine, and, unlike typhoid, a rather sudden onset, with a temperature reaching sometimes to 106 deg. in two days, is often seen. Typhoid does not take on a malarial type in certain places, as is often said, and the coincidence of the two is rare. Bastianelli[7] gives a good account of hæmoglobinuria in malaria. It occurs only in the æstivo-autumnal form. It is rare in first attacks and in very chronic forms where the body has become tolerant. It may occur spontaneously or follow quinine, but only in persons suffering or recovering from this form of ague. Quinine will in certain persons of this class bring it on whenever given, until a certain long period has elapsed. If hæmorrhage occurs in a paroxysm of malaria, and the parasites are present, he still advises quinine, but not if the parasites have disappeared from any cause. There is, however, much dissension among other authorities on the subject. Beltain[8] finds this black water fever occurs after yellow fever treated with quinine, and in other persons who have had no quinine or malaria. Dempwolf, believing it follows malaria, thinks that when the hæmoglobin is much reduced by the disease, the cells absorb quinine, which they do not do in health. W. R Livingstone[9] distinguishes mild intermittent hæmaturia, and an acute malignant type. In spontaneous outbreaks he gives quinine and urea hypodermically (Merck's preparation) in large doses, 30 and 50 grains in a day, on account of the vomiting and digestive disturbance. However, he does not seem to consider those cases where it follows the long use of quinine. When produced by quinine he does not regard it as serious, and holds that it stops with the disuse of the drug. Winslow[10] describes a case of true malaria in an infant, in whom the symptoms existed from birth. The mother was then suffering from the disease, and he suggests that it was either congenital or taken in by the milk. In either case the instance is important from its bearing on the pathology of malaria. The plasmodes were found in abundance. D. T. Caddy[11] noted a temperature of 111 deg. just before the death of a patient after two days' illness only. P. Manson,[12] by special methods of staining, has been able to demonstrate that the flagellæ arise from a central body in the crescentic amœbæ. They remain for a time curled up within the outer wall of the parasite, and are then projected outwards. Some of their free ends show a bulbous or leaf-shaped enlargement. With a weak stain, these and other details of the organism can be made out, and it seems hardly possible to regard them as a degeneration or breaking up of the amœbæ. Neil Macleod,[13] to avoid the difficulty of examining fresh films, obtains dried, unstained ones by drawing the edge of a piece of note-paper through a drop of blood not larger than a pin's head, and then again drawing the edge across a cover glass before the blood has time to dry. In this way a fine, narrow film will be produced, and quickly dries. It can be thus preserved and should be examined without any mounting, the slip being fastened down by strips of gum-paper. Sternberg[14] suggests that the normal home of the amœba may be in the stems and leaves of water-plants, whence it may get entrance not rarely into the mosquito, and he points out that when the plants are submerged by floods, malaria ceases to prevail in the neighbourhood of the swamps.

1 B. Med. Jour., May 15. 2 B. Med. Jour., July 17. 3 Lancet, Aug. 28. 4 B. Med. Jour., June 26. 5 Lancet, Oct. 2. 6 Med. Rec., March 6. 7 Am. J. Med. Sc., Aug. 8 Med. Week, July 9. 9 N. York Med. Jour. 10 Med. Rec., July 10. 11 Lancet, May 22. 12 B. Med. Jour., July 10. 13 Lancet, July 10. 14 Amer. M.S. Bullet., Ap. 10.

